# Improving stroke care in Ghana: a roundtable discussion with communities, healthcare providers, policymakers and civil society organisations

**DOI:** 10.4314/gmj.v55i2.8

**Published:** 2021-06

**Authors:** Olutobi A Sanuade, Leonard Baatiemaa, Kafui Adjaye-Gbewonyo, Ama de-Graft Aikins

**Affiliations:** 1 Institute for Global Health, University College London, London, United Kingdom; 2 Department of Health Policy, Planning and Management, School of Public Health, University of Ghana, Legon, Accra, Ghana; 3 Faculty of Education, Health and Human Sciences, University of Greenwich, London, United Kingdom; 4 Institute of Advanced Studies, University College London, London, United Kingdom

**Keywords:** stroke, care, public engagement, roundtable, collaboration

## Abstract

Even though there have been advances in medical research and technology for acute stroke care treatment and management globally, stroke mortality has remained high, with a higher burden in low- and middle-income countries (LMICs) such as Ghana. In Ghana, stroke mortality and disability rates are high, and research on post-stroke survival care is scarce. The available evidence suggests that Ghanaian stroke survivors and their caregivers seek treatment from pluralistic health care providers. However, no previous attempt has been made to bring them together to discuss issues around stroke care and rehabilitation. To address this challenge, researchers from the Institute of Advanced Studies, University College London, in collaboration with researchers from the African Centre of Excellence for Non-communicable diseases (ACE-NCDs), University of Ghana, organised a one-day roundtable to discuss issues around stroke care. The purpose of the roundtable was fourfold. First, to initiate discussion/collaborations among biomedical, ethnomedical and faith-based healthcare providers and stroke patients and their caregivers around stroke care. Second, to facilitate discussion on experiences with stroke care. Third, to understand the healthcare providers', health systems', and stroke survivors' needs to enhance stroke care in Ghana. Finally, to define practical ways to improve stroke care in Ghana.

**Funding:**

This roundtable discussion was supported by the Wellcome Trust-funded project on ‘Chronic Disease in Sub-Saharan Africa’ led by Professor Megan Vaughan [Award No: 106534]. The funders had no role in study design, data collection and analysis, decision to publish, or preparation of the manuscript.

## Introduction

Although well-established evidence of an increasing stroke burden in Ghana is evident in high mortality and disability rates,^1^,^2^ management of stroke in the healthcare settings is challenging and complex due to the pluralistic healthcare-seeking behaviours^3^,^4^ and a host of factors, in cluding poor healthcare accessibility.^4^–^6^ Existing discrepancies between explanatory models of stroke by laypeople and pluralistic healthcare providers contribute to poor stroke prognosis in Ghana.^4^,^7^ Studies have particularly shown that stroke survivors seek biomedical treatment, in addition to treatments from faith healers and herbal/traditional healers, either concurrently or sequentially.^3^,^4^ Despite this, no previous scholarly attempt has been made to report evidence of stroke care, treatment and strategies for improvement from the perspectives of policymakers, health advocates/activists, healthcare professionals as well as faith healers and herbal healers.

In light of the above context, a one-day roundtable discussion was organised on February 14, 2019, at the University of Ghana to facilitate discussion among different stakeholders around stroke care. In all, 38 participants including doctors, nurses, stroke survivors, community members, traditional healers, academia, civil society organisations (Ghana NCD Alliance and Stroke Association Support Network, Ghana) and representatives from the Ministry of Health (MOH Traditional Medicine and Alternative Practice Council) and Ghana Health Service (Non-communicable Diseases Control and Prevention Unit) participated in the discussion.

## Methods

The roundtable workshop was organised to disseminate previous studies on stroke and facilitate discussions on these findings. It was characterised by key presentations, panel discussions, group discussions and open forum. The workshop started with two presentations based on research on lived-experience of stroke in the Ghanaian setting, and nature of acute stroke care services in major referral hospitals in Ghana and the extent to which these conform to international standards.^4^,^6^ At the end of these two presentations, participants were split into three groups. Each group had representatives from different categories of attendees. The questions discussed in each group focused on three key areas, and these included: (a) experiences with stroke care; (b) major issues that Ghana is facing in the presence of the stroke burden, and (c) the way forward. These discussions were based on pre-defined questions (Table 1).

**Table 1:** Roundtable agenda, themes and questions

	Theme	Questions
**1**	Experiences with stroke care	How difficult has it been to care for people living with stroke? How does this experience compare to treating people with other illnesses? Why do people living with stroke seek different forms of treatments?
**2**	Major issues that Ghana is facing in the presence of stroke	What are the main issues Ghana is facing regarding the stroke epidemic? What are the major consequences of stroke for patients, families, communities, and healthcare providers? What have the challenges been for stroke management/care at the level of health facilities?
**3**	Looking forward	What can be done to enhance stroke management or rehabilitation in Ghana? How can healthcare professionals, faith healers and traditional/herbal healers work together to enhance preparedness for stroke rehabilitation? What roles can policymakers, telecommunication companies, NGOs, etc., play in this?

The group discussions were recorded, and notes were taken for each group. The group members provided verbal consent to the audio recordings. Key themes and analysis that emerged from the discussions are presented here, and these were based on the notes taken during the group discussions.

## Experiences with Stroke Care

### Barriers to stroke management

Consistent with prior studies,^7^,^8^ participants' discussions showed multi-level barriers to stroke management in Ghana. The barriers mentioned and discussed by participants include individual factors such as high cost of CT scans and medications, protracted medication intake, side effects of medications (including sexual dysfunction, constipation, nervousness, headache, coughing, diarrhoea), fear of the hospital, and socio-cultural beliefs which cast doubt about the potency of orthodox medicine labelled as being limited in its ability to cure a stroke; interpersonal factors (e.g. inadequate support from family caregivers); healthcare provider factors (e.g. poor attitudes of health professionals), and; health system factors (e.g. inadequate equipment such as computerised tomography (CT) scan and magnetic resonance imaging (MRI); lack of NHIS coverage for medicines; limited bed capacity). Participants' discussions revealed that these factors informed sequential and concurrent use of nonbiomedical treatments. Acknowledging and developing programmes and interventions to address the multi-layered nature of these barriers are critical to improving stroke care in Ghana.

### Caregivers' challenges with stroke care

For the caregivers, poor knowledge on causes, complications and how to care for stroke survivors was a major barrier to providing adequate care. Also, compared to caring for people living with other chronic conditions, the debilitating nature of stroke, coupled with the cognitive dysfunctions, make stroke care time consuming and physically and emotionally draining. Previous research showed that caring for stroke may be particularly difficult because of its effect on cognition and neurology, which bring about the difficulty of memory, thinking, language, attention, decision making, perception, and problem-solving.^9^ Stroke caregivers mentioned another difficulty that they experience in providing care for stroke patients. Stroke patients can be very emotional and easily angered because of the multifaceted changes in their life trajectories. This is not surprising because evidence shows that people living with stroke may experience frequent mood and emotional changes such as depression,^10^,^11^ anxiety, emotionalism, personality changes and anger due to the physical damage to their brains.^12^ As a result of these changes, stroke survivors may be overly aggressive towards their caregivers (family and health professionals).

### Health professionals' challenges with stroke care

For health professionals, inadequate medical supplies limit their ability to provide optimal stroke care. Even though they are willing to help stroke survivors, they are sometimes constrained by inadequate equipment, limited bed capacity to admit stroke patients, and difficulty getting functional CT or MRIs machines for stroke patients, which leads to delay in the start of treatment. Delay in the start of treatment often increases stroke complications. Where patients have to be moved to another health facility, many of the ambulances are ill-equipped. This sometimes means that the oxygen that is being used in the ward is moved inside the ambulance leading to deficient oxygen in the ward. Other factors discussed by health professionals include emotional instability of stroke survivors, which triggers anger and non-adherence to treatment and inadequate staffing.

## Major Issues Arising from the Burden of Stroke in Ghana

In Ghana, there is limited adoption of evidence-based acute stroke care interventions.^13^ Generally, there is inadequate equipment for stroke diagnosis, treatment and rehabilitation, and a limited health workforce for acute stroke care. A study of four tertiary hospitals and seven regional hospitals across Ghana showed that in terms of stroke workforce, four of the hospitals had neurologists, eight had clinical psychologists, one had trained stroke nurse, eight had physician specialists, three had neurosurgeons, and all of them had medical officers, nurses, emergency department staff, and physiotherapists. None of these hospitals had speech and occupational therapists.^13^ Regarding acute stroke services, treatments and rehabilitation services, only one hospital had a dedicated stroke unit.^13^

Another issue that Ghana faces regarding stroke care is the emergence of a new industry in the country focusing on commercialising adult care (including stroke care). This is due to the growing demand for elderly care providers, most especially in urban areas, to supplement the direct care of relatives who struggle with competing life and livelihood priorities in Ghana or abroad. 14 In this regard, new kinds of carers, hired through commercial nursing agencies, have emerged in the country. These carers, who are not always trained to provide complex care, ‘position themselves as professional nurses and sometimes share biomedical and scientific knowledge with clients’.^14^ The challenge with this is that these carers, who provide stroke care in some situations, are not trained as nurses. This has serious implications for stroke management and control with possibilities of poor stroke outcomes.

Participants discussed that Ghana has a pluralistic healthcare system: people seek treatment from mainstream biomedical health services and herbalists and faith healers. All these health services shape stroke care and rehabilitation in Ghana and work at cross purposes.

Also, the mainstream hospitals, which are seen as the only legitimised health system for stroke management and rehabilitation, are often under-staffed and under-resourced. Therefore, they cannot either meet the increasing number of stroke patients in Ghana or manage the long-term nature of stroke care as it is widely known that stroke is a complex clinical condition that requires a multidisciplinary team,^15^–^19^ participants mentioned that the limited number of neurologists and other key health workforce often limits the establishment of multidisciplinary teams for stroke care in Ghana. The healthcare system in the country is still structured to mostly tackle infectious diseases or not able to manage multimorbid conditions adequately.

## Recommendations on How to Improve Stroke Care

Based on the discussions, it became clear that stroke care can be improved when all of the stakeholders work together more collaboratively. Recommendations on how to improve stroke care were discussed by the participants. These were thematised based on some of the key elements of the WHO health systems' building blocks and these focused on the following: (1) leadership and governance, (2) service delivery, (3) health workforce, (4) medical products, vaccines and technologies, and (5) health information systems.^20^

### Leadership and governance

There is a need to invest in research on herbal medicines that can be used for stroke treatment. The Centre for Plant Medicine Research of the Council for Scientific and Industrial Research in Ghana can be strengthened to play a lead role. Also, it is important to intensify advocacy to create awareness of stroke warning signs. This may help to enhance early diagnosis and in turn minimise stroke complications. With the high level of religiosity in Ghana, faith-based institutions could be engaged to disseminate information on stroke warning signs and where and how to seek treatment.^21^

### Service delivery

Participants highlighted that it is important for health professionals to create an enabling environment where stroke survivors can freely discuss their illness. This was raised as a critical issue that needs to be addressed to enhance doctor-patient trust and ensure that treatment strategies are centred on patients' needs. Socio-cultural explanations about the causes of stroke, as discussed by the stroke survivors and caregivers, should be considered during care delivery. Ignoring this may hinder medication adherence. Further, there is a need to develop a model for the long-term management of stroke in Ghana.

Due to limited stroke units and the cost of treatment, many stroke survivors are being cared for in their respective communities. Therefore, community interventions to address long-term stroke care need to be initiated. This can be done through a task-shifting approach that trains community health workers to deliver stroke care. Evidence in Ghana has shown that well-trained community health workers can adequately manage uncomplicated cardiovascular disease.^22^

### Health workforce

Participants mentioned the need to increase the number of trained health professionals to deliver stroke care and rehabilitation. This was based on the premise that the number of trained stroke workforce in Ghana is low.

### Medical products, vaccines, and technologies

The need for policymakers to expand NHIS coverage to cover stroke medications and treatments was raised as an important issue by all stakeholders. This can increase medication adherence among patients and minimise catastrophic health expenditure. Participants also emphasised the importance of having mobile stroke units in Ghana. This will increase early diagnosis and treatment of acute stroke. In the meantime, it is important to ensure that ambulances are well equipped with the necessary equipment to help stabilise stroke patients before being admitted to the hospital.

### Health information systems

Participants highlighted the need to generate context-specific or local empirical evidence on stroke burden instead of relying on international documents or modelled estimates. This will ensure that care is consistent with stroke patients and local needs. It will also help build the evidence base to determine all the resources and equipment needed for stroke care and rehabilitation in Ghana.

## Outcome

Generally, there was a discussion around the fact that stroke care is a daunting task that requires an ‘all-hands-on-deck approach’, involving health professionals, traditional practitioners, faith healers, caregivers and other family members, civil societies, and the entire community. Since hospitals are limited in their ability to provide long-term care for stroke survivors, it was suggested that while the sudden onset of stroke requires attention from the biomedical practitioners as the first point of contact, registered community herbal healers fill an important niche in stroke and management in long-term care. Some participants mentioned that biomedical practitioners can focus on the acute care of stroke while they discharge patients to some of the registered community herbal clinics with constant supervision. Whether or not these healers have the acclaimed capability demands further study.

Nevertheless, existing research showed that although herbal clinics offer consultations and produce phytotherapeutic medicines in their laboratories, organisation of their workplaces, hygiene and standardisation are often inadequate. In addition, their records and description of their activities often suggest amateurism.^23^

This roundtable workshop showed that the importance of using task-shifting approaches to deliver stroke care at the community level in Ghana could not be overemphasised. It is important to note that the tension between the various health practitioners in Ghana is not only seen in stroke care but the management of non-communicable disease (NCD) in general. Nevertheless, this workshop laid the important groundwork by offering insights for reducing cross-professional tensions and promoting collaborations among different health stakeholders in the country.
